# MRI and high-resolution ultrasound of plantar plate pathology in the painful forefoot of patients with Rheumatoid Arthritis

**DOI:** 10.1186/1757-1146-3-S1-O24

**Published:** 2010-12-20

**Authors:** Heidi Siddle, Anthony Redmond, Richard Wakefield, Richard Hodgson, Andrew Grainger, David Pickles, Philip Helliwell

**Affiliations:** 1 University of Leeds, Leeds, UK; 2 Leeds Musculoskeletal Biomedical Research Unit, Leeds, UK; 3 Leeds Teaching Hospitals NHS Trust, Leeds, UK

## Introduction

Forefoot pain in Rheumatoid Arthritis (RA) is associated with functional and structural changes at the MTP joints. Previous cadaveric studies have suggested that forefoot deformities in RA might result from the failure of a complex ligamentous system and displacement of the plantar plates (PP). MRI and high resolution ultrasound (HRUS) have been reported to identify the PP and visualise tears in otherwise healthy subjects.

## Methods

In 24 patients with RA the more symptomatic forefoot was imaged using MRI and HRUS. The images were assessed to determine the presence or absence of the PP and to identify tears and their location.

## Results

17 females and 7 males with a mean (SD) age of 55.5 (10.5) years and disease duration 10.6 (8.6) (range 0.6 - 36) years took part in the study. The mean (SD) VAS forefoot pain score was 43.4 (27.9).

Tears in the lesser PP were identified in 18 (75%) patients on MRI. 4 tears were observed in the 2^nd^ PP, 5 in the 3^rd^, 9 in the 4^th^ and 13 in the 5^th^. All tears were located distally, 6 (19.4%) were full width tears and the majority (58.1%) of partial tears were situated medially. Tears were not identified on HRUS (Table 1).

**Table 1:** 

	Presence of plantar plates			# absent plantar plates by site			
	All present	≥ 1 plate absent	All absent	MTP2	MTP3	MTP4	MTP5

MRI	14	9	1	2	3	3	8
HRUS	10	13	1	2	3	8	9

## Discussion

In patients with RA and forefoot pain MRI demonstrates that PP tears are common at the lesser MTP joints, particularly the 5th. The reporting of absence of plantar plates was consistent for HRUS and MRI findings except at the 4th MTP joint, deformity may result in difficulty imaging this joint with HRUS. This study suggests that forefoot pain and deformity in RA is associated with PP pathology although does not establish causality. PP pathology has been well demonstrated with MRI, however contrary to recent research in normal subjects there is a discrepancy between MRI and HRUS in detecting tears in the PP of the lesser MTP joints in patients with RA.

